# 26 cm fall caught on video causing subdural hemorrhages and extensive retinal hemorrhages in an 8‐month‐old infant

**DOI:** 10.1002/ccr3.9105

**Published:** 2024-06-25

**Authors:** Chris Brook, Waney Squier, Julie Mack

**Affiliations:** ^1^ Universidad de La Laguna, Avda. Astrofísico Fco. Sánchez La Laguna Spain; ^2^ Previously Department of Neuropathology John Radcliffe Hospital Oxford UK; ^3^ Radiology, Penn State Hershey Medical Center Hershey Pennsylvania USA

**Keywords:** abusive head trauma, retinal hemorrhages, shaken baby syndrome, short falls

## Abstract

Severe, too many to count retinal hemorrhages (RH) in infants have been associated with abusive head trauma, but can occur in short falls. An 8‐month‐old male fell backward from a height of 26 cm, landing on his buttocks then hitting the back of his head on a vinyl floor. The fall was videotaped. Acute subdural hemorrhages were found along with extensive, too many to count intra‐RH in both eyes. Falls from small heights on to the occiput can lead to extensive RH of the type often associated with abusive head trauma.

## 1 INTRODUCTION

Since the seminal publication of Plunket,^1^ case studies^2^, ^3^, ^4^, ^5^, ^6^, ^7^, ^8^, ^9^, ^10^, ^11^ have continued to demonstrate that short falls can cause subdural hemorrhages (SDH) and extensive retinal hemorrhages (RH). While cases with independently witnessed short falls are important sources of evidence, falls caught on video are even more compelling. Because filmed cases are rare, it is important that they are documented in the literature. Here we report on the videotaped short fall of an alert, healthy and active infant from a low sofa that led to seizures, acute SDH and extensive RH.

## 2 CASE HISTORY

The male infant was 8 months old, born via normal vaginal delivery at 39 + 4 weeks gestation, weighing 3.6 kg. Vitamin K and hepatitis B injections were given at birth. One month prior to the fall, the mother took the infant to hospital reporting twitching of one shoulder and arm. The infant was assessed and sent home with no investigations or treatment. Roseola was diagnosed 2 weeks prior to hospitalization, which had resulted in fever and rash.

The head circumference was 47 cm (99th percentile) 1 month prior to the fall, and 47.6 cm (99th centile) 1 day after the fall.

Two days prior to the fall, the infant had vomited and was eating less than usual. The mother reported that the infant was learning to walk and “furniture surf” so had falls most days. The infant had fallen forward off a low trampoline 15 cm off the ground onto concrete resulting in an abrasion of his nose a few days prior to the videotaped fall.

On the day of the fall, the mother left the infant at a gym creche, at which time he was alert, active, fully responsive, and (at least outwardly) healthy. The infant sat on his bottom on the edge of the couch and fell backward, with his bottom hitting the floor and his occiput hitting the floor immediately thereafter. The distance from the ground to the seat of the couch is 26 cm, while head to ground height is approximately 65–70 cm prior to the fall. The fall was caught on the surveillance video of the creche (see Video S1). There is a rotational component to the accident, with the buttocks hitting first and acting as a pivot point. The body then rotates around an axis passing through the hips, as the infant falls backward until the back of head hits the floor.

After the fall the mother collected the infant, at which time he was drowsy, “a bit floppy” and unresponsive. Once taken to the car, the mother reported a 5 s episode of arm and leg stiffening, back arching and eye deviations upwards and to the right. She took him to the local hospital from where he was transferred to a children's hospital.

### 2.1 Clinical presentation

**FIGURE 1:**
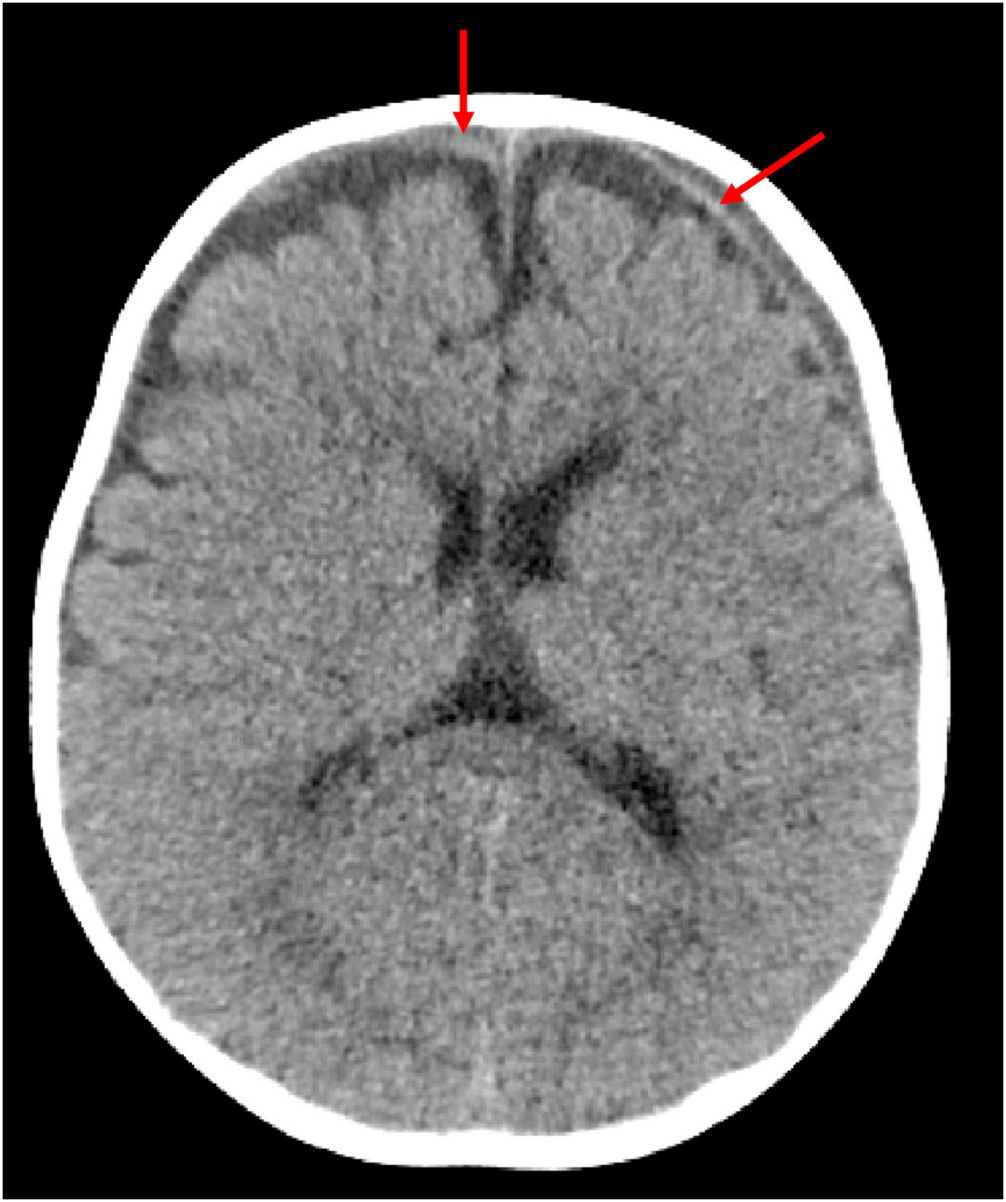
Axial image non‐contrast CT brain showing hyperdense (acute) linear subdural collection on the left with associated hypodense subdural fluid (chronic collection). There is a smaller hyperdense collection on the right.

**FIGURE 2:**
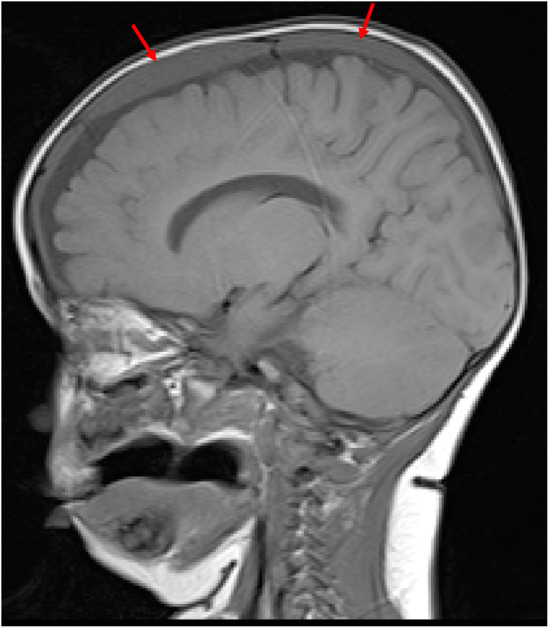
T1 weighted sagittal MRI 7 days after the fall injury. There is a holohemispheric isointense subdural collection present on the left. There is a smaller collection on the right (not shown).

A CT brain on the day of the accident showed bilateral small volume acute, high density, SDH on a background of chronic, low density, collections (Figure 1). The child was kept in hospital and monitored. Seven days later, an MRI showed bilateral subdural collections measuring up to 5 mm thick without significant mass effect (Figure 2). The extra axial CSF spaces and lateral ventricles were judged to be normal. No parenchymal diffusion abnormalities were identified. Midline structures and cranio‐cervical junction were normal.

It was noted that dating the SDHs was inaccurate due to dilution of blood products. The acute SDHs were attributed to the fall.

**FIGURE 3:**
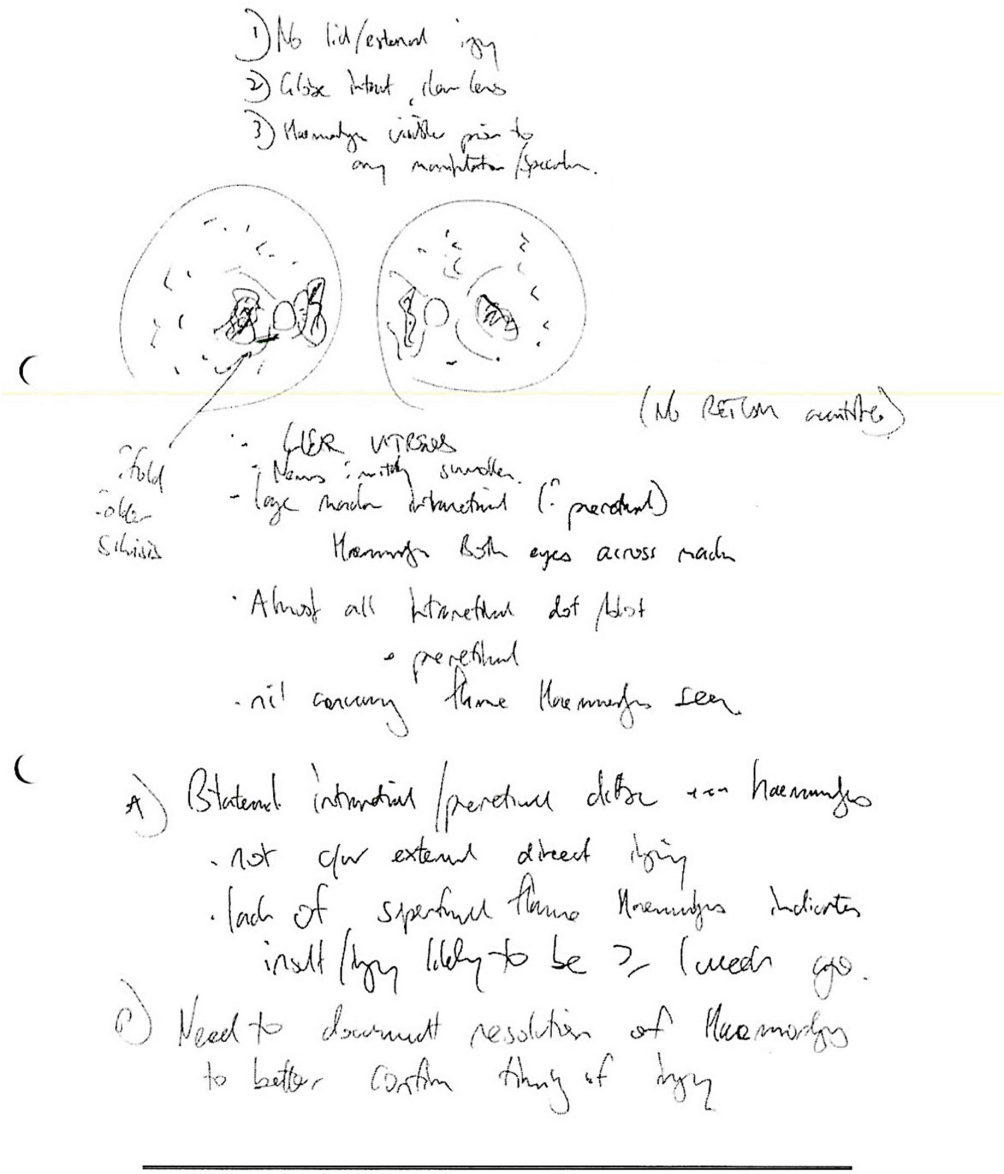
Drawing of the retinal hemorrhages.

Less than 24 h after the fall, eye examination was performed without ocular manipulation. Binocular indirect ophthalmoscope with 30D lens found multiple RH in both eyes, too numerous to count (at least >30 in each eye), large intra‐retinal macular hemorrhages extending to retinal periphery in all quadrants in each eye and a retinal fold along the inferior arcade of the right eye. Almost all RHs in both eyes were intra‐retinal dot/blot as well as preretinal. The optic nerves in each eye were mildly swollen. No RetCam images were taken, but the ophthalmologist made sketches (Figure 3).

Seven days after the fall, the ophthalmology team re‐examined the eyes and noted bilateral intra‐retinal/ large preretinal diffuse hemorrhages. The RH were resolving slowly, as expected.

Intra retinal hemorrhages (IRH) have been shown to resolve rapidly, within 1–2 weeks clearing much faster than preretinal hemorrhages.^12^, ^13^, ^14^ Too numerous to count (>20) IRHs can resolve in a matter of days.^14^

In this case, there were too numerous to count IRHs at first examination, <24 h after the fall. The IRHs resolved slowly, still present 1 week later. This indicates that the RHs occurred around the time of the fall and, given the changes to the state of the infant caused by the fall, it is reasonable to attribute the RHs to the fall.

There were no external signs of injury: no bruising and a skeletal survey and nuclear medicine bone scans demonstrated no evidence of fractures.

No abnormality was demonstrated at cranio‐cervical junction. The spinal cord appeared normal in signal and in contour. No structural abnormality of the spinal cord was identified.

**FIGURE 4:**
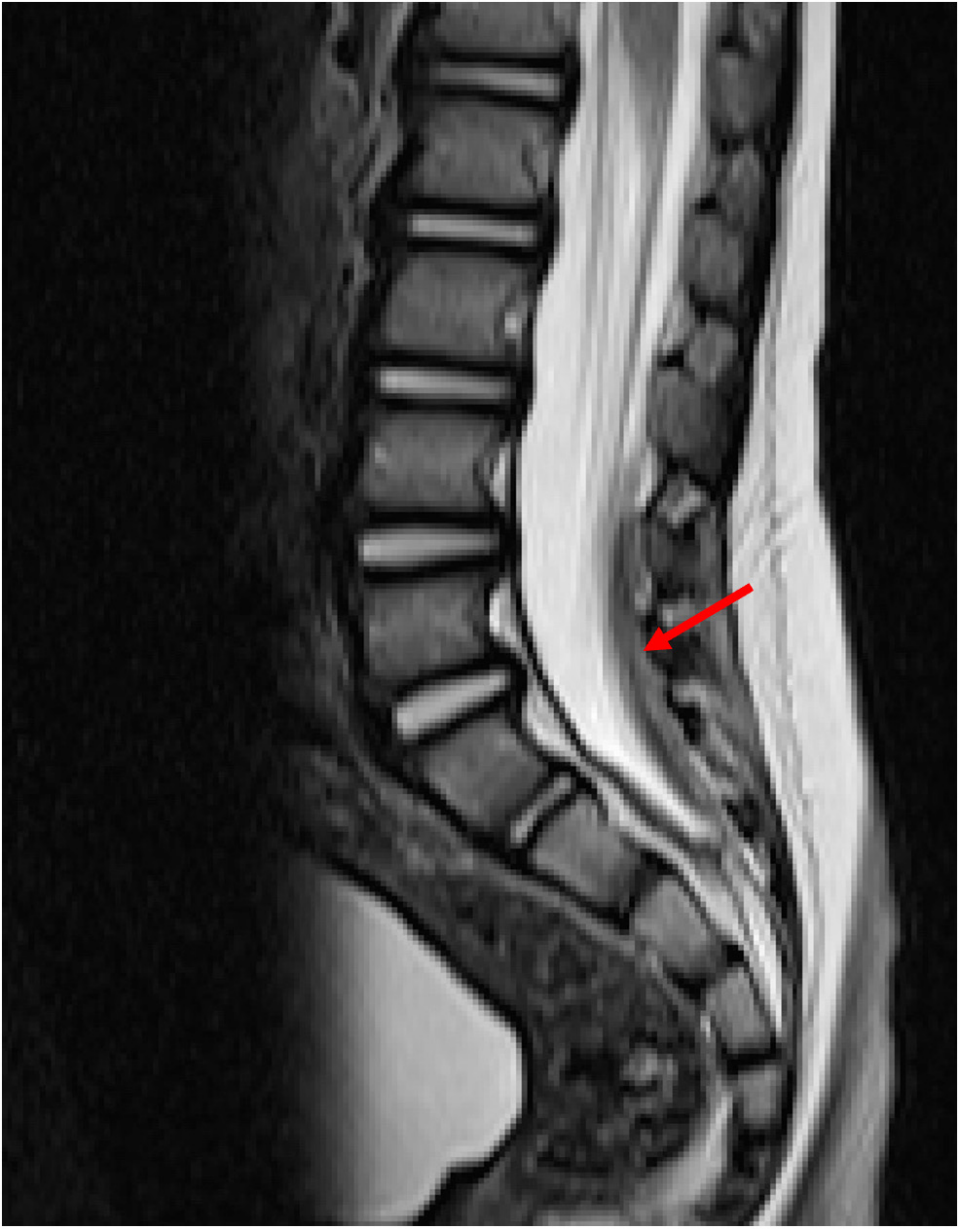
MRI spine 7 days after the fall shows a small subdural collection layering dependently in the lumbo‐sacral region.

There was a small subdural collection in the lumbosacral spine that was isointense on T1 and hypointense on T2 (Figure 4). There were no signal changes within the vertebral bodies to suggest occult compression fracture and no other abnormality was detected (no soft tissue edema).

The infant improved rapidly and was neurologically normal the day after the fall.

### 2.2 Outcome

The child was removed from the parents, along with two siblings, based on the advice of a child abuse pediatrician that the findings were due to abusive head trauma.

After an investigation it was determined that the fall caused the hemorrhages, and no charges were laid. After 12 months separation, the children were returned to the family. This highlights the potential harm resulting from determinations of child abuse based on findings that can have non‐abusive origins. The trauma caused by parent–child separation, which has been shown to be harmful to infants,^15^ is also traumatic for parents.

## 3 DISCUSSION

This case adds to previous studies that have shown that short falls can result in SDHs and RHs. Low level falls onto the occiput have been associated with extensive RHs and SDH.^6^, ^11^ It is of note that most babies in the Atkinson series, like the present case, had large heads circumference (>90th percentile).

Mattheij^16^ also found that infants with a large head circumference could be predisposed to RH or SDH. In a study of 29 babies with RHs, of whom 6 had no suspicion of abusive head trauma, they found “no differences between the groups concerning the location, distribution, or size of the RH” leading the authors to conclude that “there is no pathognomonic size, distribution, or location of RH seen only in AHT”.^16^

Recently, Sokoloff et al.^17^ categorized cases with loss of consciousness (LOC) and/or bilateral/interhemispheric SDH (bihSDH) as involving inertial forces, linking them to extensive RHs. While they cited studies suggesting that inertial forces can cause LOC and/or bihSDH, that does not rule out other causes like contact injury. Thus, it remains unclear whether contact injuries alone can cause LOC and/or bihSDH and hence be associated with extensive RHs, or whether intertial forces are required.

In this context, it is interesting that the fall in the current case involved both contact and intertial (rotational) forces, and that both contact and intertial forces were also identified in another recent short fall case that was videotaped,^10^ and which also had SDH and extensive RHs.

It is also worth noting that the mother reported prior low height falls in this infant. Prior traumatic brain injury has been shown to be a predisposition to more severe subsequent traumatic brain injury.^18^, ^19^

## 4 CONCLUSION

A short, low impact, occipital fall on a vinyl floor was captured on video. The fall, which involved both contact and rotational forces, resulted in a changed state of consciousness, acute SDHs and extensive, too many to count RH that extended to the periphery in both eyes. The baby had macrocrania and possible chronic SDH which may have accounted for the severe RH rather than the fall, or may have made the infant particularly vulnerable to trauma.^16^

## AUTHOR CONTRIBUTIONS

**Chris Brook:** Conceptualization; writing – original draft. **Waney Squier:** Conceptualization; writing – review and editing. **Julie Mack:** Formal analysis; visualization.

## FUNDING INFORMATION

The authors have no financial relationships relevant to this article to disclose.

## CONFLICT OF INTEREST STATEMENT

CB declares no conflicts of interest. WS has testified in court regarding SBS cases, both for the defense and the prosecution. JM has testified, unpaid, for the defense regarding SBS cases.

## CONSENT

Written informed consent was obtained from the patient to publish this report in accordance with the journal's patient consent policy.

## Supporting information


Video S1.

## Data Availability

Data sharing not applicable—no new data generated.

## References

[1] Plunkett J . Fatal pediatric head injuries caused by short‐distance falls. Am J Forensic Med Pathol. 2001;22:1‐12. 11444653 10.1097/00000433-200103000-00001

[2] Denton S , Mileusnic D . Delayed sudden death in an infant following an accidental fall: a case report with review of the literature. Am J Forensic Med Pathol. 2003;24(4):371‐376. 14634479 10.1097/01.paf.0000097851.18478.16

[3] Goldsmith W , Plunkett J . Biomechanical analysis of the causes of traumatic brain injury in infants and children. Am J Forensic Med Pathol. 2004;25:89‐100. 15166757 10.1097/01.paf.0000127407.28071.63

[4] Gardner HB . A witnessed short fall mimicking presumed shaken baby syndrome (inflicted childhood neurotrauma). Pediatr Neurosurg. 2007;43(5):433‐435. doi:10.1159/000106399 17786015

[5] Scheller J , Huisman TAGM . Moderate bilateral retinal hemorrhages in an infant following a short fall. Clin Pediatr. 2015;54(10):999‐1002. doi:10.1177/0009922814554501 25316220

[6] Atkinson N , van Rijn R , Starling S . Childhood falls with occipital impacts. Pediatr Emerg Care. 2018;34(12):837‐841. 28590993 10.1097/PEC.0000000000001186

[7] Aoki N . Infantile acute subdural hematoma with retinal hemorrhage caused by minor occipital impact witnessed by an ICU nurse: a case report. J Pediatr Neurol Neurosci. 2020;4(1):47‐50.

[8] Raj A , Christian CW , Reid JE , Binenbaum G . A baby carrier fall leading to intracranial bleeding and multilayered retinal hemorrhages. J AAPOS. 2022;26(2):84‐86. doi:10.1016/j.jaapos.2021.10.008 35091083

[9] Aoki N . Clinical and neuroimaging characteristics in mild‐type infantile acute subdural hematoma: a report of four cases. Research Square Platform LLC. 2023. doi:10.21203/rs.3.rs-2733567/v2 PMC10761517 37581738

[10] Geoghegan AR , Shouldice M , Mireskandari K , Smith JN . Subdural hemorrhages and severe retinal hemorrhages in a short fall with a rotational component. J AAPOS. 2023;27:222‐224. 37307907 10.1016/j.jaapos.2023.04.011

[11] Kato M , Nonaka M , Akutsu N , Narisawa A , Harada A , Park Y‐S . Correlations of intracranial pathology and cause of head injury with retinal hemorrhage in infants and toddlers: a multicenter, retrospective study by the J‐HITs (Japanese head injury of infants and toddlers study) group. PLoS One. 2023;18(3):e0283297. doi:10.1371/journal.pone.0283297 36930676 PMC10022784

[12] Hughes LA , May K , Talbot JF , Parsons MA . Incidence, distribution, and duration of birth‐related retinal hemorrhages: a prospective study. J AAPOS. 2006;10:102‐106. 16678742 10.1016/j.jaapos.2005.12.005

[13] Watts P , Maguire S , Kwok T , et al. Newborn retinal hemorrhages: a systematic review. J AAPOS. 2013;17:70‐78. 23363882 10.1016/j.jaapos.2012.07.012

[14] Binenbaum G , Chen W , Huang J , Ying GS , Forbes BJ . The natural history of retinal hemorrhage in pediatric head trauma. J AAPOS. 2016;20(2):131‐135. doi:10.1016/j.jaapos.2015.12.008 27079593 PMC4839593

[15] Hofer MA . Psychobiological roots of early attachment. Curr Dir Psychol Sci. 2006;15(2):84‐88.

[16] Mattheij M , Venstermans C , de Veuster I , et al. Retinal haemorrhages in a university hospital: not always abusive head injury. Acta Neurol Belg. 2017;117(2):515‐522. doi:10.1007/s13760-017-0748-0 28160241

[17] Sokoloff M , Feldman KW , Levin AV , et al. Retinal hemorrhage variation in inertial versus contact head injuries. Child Abuse Negl. 2024;149:106606. doi:10.1016/j.chiabu.2023.106606 38134727

[18] Raghupathi R , Margulies SS . Traumatic axonal injury after closed head injury in the neonatal pig. J Neurotrauma. 2002;19(7):843‐853. 12184854 10.1089/08977150260190438

[19] Corrigan F , Mander KA , Leonard AV , Vink R . Neurogenic inflammation after traumatic brain injury and its potentiation of classical inflammation. J Neuroinflammation. 2016;13:264. doi:10.1186/s12974-016-0738-9 27724914 PMC5057243

